# SARS-CoV-2 Membrane Glycoprotein M Triggers Apoptosis With the Assistance of Nucleocapsid Protein N in Cells

**DOI:** 10.3389/fcimb.2021.706252

**Published:** 2021-08-25

**Authors:** Yujie Ren, An Wang, Yuan Fang, Ting Shu, Di Wu, Chong Wang, Muhan Huang, Juan Min, Liang Jin, Wei Zhou, Yang Qiu, Xi Zhou

**Affiliations:** ^1^Guangzhou Institute of Pediatrics, Guangzhou Women and Children’s Medical Center, Guangzhou, China; ^2^State Key Laboratory of Virology, Wuhan Institute of Virology, Center for Biosafety Mega-Science, Chinese Academy of Sciences (CAS), Wuhan, China; ^3^Joint Laboratory of Infectious Diseases and Health, Wuhan Institute of Virology & Wuhan Jinyintan Hospital, CAS, Wuhan, China; ^4^College of Life Sciences, University of Chinese Academy of Sciences, Beijing, China; ^5^Institute of Microbiology, Jiangxi Academy of Sciences, Nanchang, China

**Keywords:** SARS-CoV-2, nucleocapsid protein, apoptosis, PDK1-Akt signaling, membrane glycoprotein

## Abstract

The pandemic of COVID-19 by SARS-CoV-2 has become a global disaster. However, we still don’t know how specific SARS-CoV-2-encoded proteins contribute to viral pathogenicity. We found that SARS-CoV-2-encoded membrane glycoprotein M could induce caspase-dependent apoptosis *via* interacting with PDK1 and inhibiting the activation of PDK1-PKB/Akt signaling. Our investigation further revealed that SARS-CoV-2-encoded nucleocapsid protein N could specifically enhance the M-induced apoptosis *via* interacting with both M and PDK1, therefore strengthening M-mediated attenuation of PDK1-PKB/Akt interaction. Furthermore, when the M-N interaction was disrupted *via* certain rationally designed peptides, the PDK1-PKB/Akt signaling was restored, and the boosting activity of N on the M-triggered apoptosis was abolished. Overall, our findings uncovered a novel mechanism by which SARS-CoV-2-encoded M triggers apoptosis with the assistance of N, which expands our understanding of the two key proteins of SARS-CoV-2 and sheds light on the pathogenicity of this life-threatening virus.

## Introduction

Coronavirus Disease 2019 (COVID-19) pandemic caused by severe acute respiratory syndrome coronavirus 2 (SARS-CoV-2) has become the worst public health crisis once a century. COVID-19 symptoms range from mild to severe and critical illness, including acute respiratory distress syndrome (ARDS) and life-threatening sepsis (Arshad Ali et al., 2020). Although extensive efforts have been made to fight COVID-19 and SARS-CoV-2, the underlying molecular mechanisms of its pathogenesis, particularly how individual SARS-CoV-2-encoded proteins contribute to viral pathogenicity, are still quite limited.

SARS-CoV-2 belongs to the genus *Betacoronavirus* in the family of *Coronaviridae* (de Wit et al., 2016), which also contain SARS-CoV and Middle East respiratory syndrome coronavirus (MERS-CoV), both of which cause severe respiratory diseases and the epidemic in 2002 and 2012, respectively (Arabi et al., 2015). Typical coronaviruses contain a single-stranded, positive-sense RNA genome of 26-31 kb in length and encode at least 6 open reading frames (ORFs). The first two-thirds of the coronavirus genome contains 2 ORFs, ORF1a and ORF1b, which are translated into two large polyproteins, pp1a and pp1ab, which are proteolytically processed into 15 or 16 non-structural proteins (Almazan et al., 2014). The remaining one-third encodes for the structural proteins such as the spike (S), envelope (E), membrane (M), and nucleocapsid (N), and the interspersed accessory proteins (Chen and Guo, 2016). Previous studies on SARS-CoV and MERS-CoV found that the structural proteins play critical roles in virus-host interactions and/or regulating host immune responses, contributing to the pathogenicity of coronaviruses (Frieman et al., 2008). However, little is known about the functions of SARS-CoV-2-encoded structural proteins, which impedes the understanding of the pathogenicity of SARS-CoV-2 and COVID-19.

Apoptosis is a major type of programmed cell death triggered by mitochondrion-mediated (intrinsic) or cell-surface death receptor-mediated (extrinsic) process involving the cleavage of a group of cysteinyl aspartate proteases (caspases) cleavage (activation) (Hengartner, 2000; Schultz and Harrington, 2003; Bagci et al., 2013). The activated death receptors trigger the extrinsic pathway, resulting in the activation of downstream initiator caspases-8/10 and effector caspases-3/7. The intrinsic pathway activated by intracellular signals leads to the accumulation of cytochrome c in the cytosol and induces the formation of apoptosome and caspase-9 activation and the activation of effector caspases-3 and -7 (Putcha et al., 2002). The physiological roles of apoptosis in pathogen infection are complicated. On the one hand, apoptosis is considered an efficient antiviral defense since it eliminates infected and damaged cells. Pathogen-induced apoptosis in host cells, on the other hand, would facilitate their infection and contribute to viral pathogenicity (Benedict et al., 2002). Many viruses have evolved to encode pro-apoptotic proteins that target the crucial components within the apoptotic cascade to induce apoptosis. In addition, virus-induced apoptosis is tightly linked to viral pathogenesis.

The PDK1 (3-phosphoinositide-dependent protein kinase-1)-PKB/Akt (protein kinase B) signaling pathway plays important anti-apoptotic roles (Putcha et al., 2002). PDK1 is a serine/threonine kinase that phosphorylates many protein kinases *via* an N-terminal KD (kinase domain) and a C-terminal PH (pleckstrin homology) domain (Mora et al., 2004; Manning and Cantley, 2007). PKB/Akt is a serine/threonine kinase that may be phosphorylated and activated by PDK1 and, in turn, phosphorylates and inhibits its downstream substrates, including FKHRL1 (forkhead transcription factor) and ASK (apoptosis signal-regulating kinase) (Najafov et al., 2012). The phosphorylated form of FKHRL1 is retained in the cytoplasm, but when the kinase activity of PKB/Akt is compromised, FKHRL1 translocates to the nucleus and subsequently promotes the expression of the pro-apoptotic gene FasL (Fas ligand), which cause caspase-8 activation (Brunet et al., 1999; Suhara et al., 2002; Kavurma and Khachigian, 2003). Meanwhile, the compromised activity of PKB/Akt also results in the decreased phosphorylation of ASK, which eventually leads to caspase-9 activation (Kim et al., 2001; Dhanasekaran and Reddy, 2008). Thus, the disruption of PDK1-PKB/Akt signaling resulted in apoptosis *via* caspase activation.

Previous studies have shown that both M and N of SARS-CoV show the activity to induce apoptosis (Surjit et al., 2004; Tsoi et al., 2014). This study found that, unlike SARS-CoV analogs, SARS-CoV-2-encoded M but not N possesses the pro-apoptotic activity that depends on the caspase cascade *via* PDK1-PKB/Akt signaling. Moreover, although SARS-CoV-2 N does not directly induce apoptosis, it can enhance the apoptosis induced by SARS-CoV-2 M by strengthening the interaction between M and PDK1, which suppresses PDK1-PKB/Akt interaction and downstream signaling. Furthermore, we demonstrate that disrupting the M-N interaction by some rationally designed peptides can effectively inhibit N’s boosting impact on the M-induced apoptosis. Our study uncovers a novel mechanism by which SARS-CoV-2 M triggers apoptosis, with N serving as a critical cofactor for M’s pro-apoptotic action.

## Methods

### Cell Culture and Transient Transfection

Cell culture and transient transfection were described previously (Lu et al., 2017; Zhou et al., 2020). In brief, Vero E6 cells, HEK393T cells, and HepG2 cells were cultured in DMEM containing 10% FBS (Gibco) and 1% streptomycin-penicillin (Gibco) at 37°C in a 5% CO_2_ incubator. According to the manufactory’s instruction, cells were seeded onto the dish 24 h prior to transfection, and plasmids were transiently transfected using TransEasyTM Transfection Reagent (Foregene).

### Reagents and Antibodies

Caspase-8 inhibitor (MCE); caspase-9 inhibitor (MCE); general caspase inhibitor (MCE); STS (Selleck); anti-cytochrome *c* (Abcam); anti-glutamate dehydrogenase (GDH; CST); HRP-conjugated goat-anti mouse or rabbit IgG (Thermo Scientific); anti-β-Actin (Sigma); anti-caspase-8 (TransGen Biotech *ProteinFind*); anti-cleaved-caspase-8 (CST); anti-caspase-9 (TransGen Biotech *ProteinFind*); anti-cleaved-caspase-9 (CST); anti-FLAG (Sigma); anti-ASK (CST); anti-p-ASK (CST); anti-FKHRL1 (CST); anti-p-FKHRL1 (CST); anti-PKB/Akt (CST); anti-p-PKB/Akt (CST); anti-GFP (Sigma), ECL(4A Biotech).

### Plasmid Construction

Plasmid construction was described previously (Lu et al., 2017; Shu et al., 2020). In brief, we got the clones of SARS-CoV-2 M, SARS-CoV-2 N, PDK1, and Akt through the combination of whole genes. The clones were digested with restriction enzymes (SalI and NotI, Thermo) and ligated to the PRK vectors (with FLAG, HA, or GFP tag) with T4 ligase (Thermo). The wild type PRK-SARS-CoV-2 M, PRK-SARS-CoV-2 N, and PRK-PDK1 constructs were used as a template to generate the mutant constructs. All mutations were confirmed by DNA sequencing.

### Immunofluorescence Staining Assays

Cells seeded on confocal dish were transfected with 1μg indicated plasmids. At 24h after transfection, cells were washed three times with PBS and fixed with 3.7% formaldehyde in PBS for 30min, then permeabilized by 1% Triton X-100 in PBS for 20min. After blocking with 5% BSA in PBS for 60 min, cells were incubated with mouse anti-FLAG antibody and anti-cleaved-caspase-3 diluted in 1:100 in 5% BSA in PBS at 4°C overnight. After washing three times with PBS, cells were incubated with Alexa Fluor 488 goat anti-mouse IgG (H+L) (abcam) and Cy3 Goat Anti-Rabbit IgG (H+L) (ABclonal) diluted in 1:500 in 5% BSA in PBS for 30 min. After washing cells three times with PBS, cell nuclei were stained with DAPI (Invitrogen) diluted in 1:250 in PBS for 5 min. Fluorescent signals were detected by using a Nikon microscope and images were analyzed with the NIS-Elements Viewer 4.50.

### Western Blot and Co-Immunoprecipitation Assays

Western blot assays were described previously (Ren et al., 2016). In brief, at the indicated times post-transfection, cells were scraped then lysed in cell lysis buffer [20 mM Tris/HCl (pH 8), 150 mM NaCl, 1% Triton X-100, 1 mM EDTA, 1 mM EGTA, 1 mM NaF, 1 mM sodium orthovanadate and protease inhibitor cocktail (Selleck)] and clarified by centrifugation at 12000 g for 15 min at 4°C. For co-immunoprecipitation, the cell lysate was incubated with protein A/G and indicated antibodies at 4°C for 2 h. For direct analysis of protein expression, the protein concentration of the lysate was incubated with loading buffer for 10 min at 95°C. Then the samples were separated with SDS polyacrylamide gel electrophoresis (SDS-PAGE).

### Mitochondria Fractionation Assays

At the indicated times post-transfection, cells were washed with PBS followed by dousing 20 times in 1 mL homogenization buffer (ApplyGen) by 1mL injector. The homogenate was centrifuged at 500 g for 10 min. The supernatant was centrifuged at 5000 g for 10 min to precipitate mitochondria.

### Apoptosis Detection Assays

Flow cytometry assay was used to detect cell apoptosis and analyze the cell cycle (Ding et al., 2019). Cells seeded on the dish were transfected with designated plasmids or treated with different caspase inhibitors (Targetmol) or caspase inducers (MCE). After 24 h, cells were washed three times with PBS and collected by centrifugation at 1500 g for 5 min, following used the “Annexin V-FITC Apoptosis Detection Kit (Beyotime)” to detect cell apoptosis. The operation steps are briefly described as follows: Add 195 μL Annexin V-FITC binding solution to resuspend cells gently; Then add 5 μL Annexin V-FITC and 10 μL propidium iodide staining solution and mix gently; Incubate at room temperature (20-25°C) in the dark for 10-20 minutes, then place in an ice bath; Flow cytometry analysis within 1 h.

## Results

### SARS-CoV-2 M Induces Caspase-Dependent Apoptosis, While N Specifically Enhances M-Induced Apoptosis.

**Figure 1:**
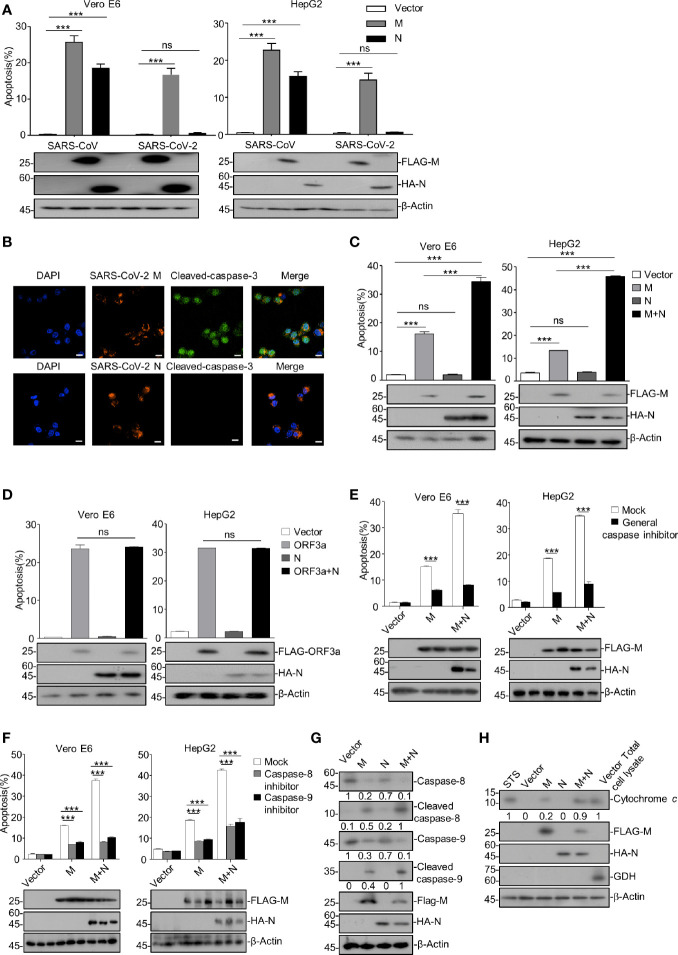
SARS-CoV-2 M induces caspase-dependent apoptosis, and N specifically enhances M-induced apoptosis. **(A)** Vero E6 and HepG2 cells were transfected with FLAG-SARS-CoV-2 M (M), HA-SARS-CoV-2 N (N), and FLAG-SARS-CoV-2 M plus HA-SARS-CoV-2 N (M+N), also with the counterparts of SARS-CoV. After 24 h, cells were stained with Annexin V-FITC/PI for flow cytometry analysis, and the percentage of apoptotic cells was measured. **(B)** Vero E6 cells were transfected with FLAG-SARS-CoV-2 M or FLAG-SARS-CoV-2 N, respectively. After 24 h, cells were processed for immunofluorescence and co-stained with anti-FLAG and anti-cleaved-caspase-3 antibodies indicated. Scale bar, 20 μm. **(C)** Vero E6 and HepG2 cells were transfected with FLAG-SARS-CoV-2 M (M), HA-SARS-CoV-2 N (N), and FLAG-SARS-CoV-2 M plus HA-SARS-CoV-2 N (M+N). After 24 h, cells were collected for Western blotting (with anti-FLAG, anti-HA, or anti-β-Actin) or stained with Annexin V-FITC/PI for flow cytometry analysis, and the percentage of apoptotic cells were measured. **(D)** Vero E6 and HepG2 cells were transfected with FLAG-SARS-CoV-2 ORF3a (ORF3a), HA-SARS-CoV-2 N (N), and FLAG-SARS-CoV-2 ORF3a plus HA-SARS-CoV-2 N (ORF3a+N). After 24 h, cells were collected for Western blotting (with anti-FLAG, anti-HA, and anti-β-Actin) or stained with Annexin V-FITC/PI for flow cytometry analysis, and the percentage of apoptotic cells were measured. **(E, F)** Vero E6 and HepG2 cells were transfected with FLAG-SARS-CoV-2 M and FLAG-SARS-CoV-2 M plus HA-SARS-CoV-2 N and treated with DMSO, a general caspase inhibitor, a caspase-8 inhibitor, and caspase-9 inhibitor, respectively. After 24 h, cells were collected for Western blotting (with anti-FLAG, anti-HA, or anti-β-Actin) or stained with Annexin V-FITC/PI for flow cytometry analysis, and the percentage of apoptotic cells were measured. **(G, H)** HEK293T cells were transfected with FLAG-SARS-CoV-2 M, HA-SARS-CoV-2 N, and FLAG-SARS-CoV-2 M plus HA-SARS-CoV-2 N (STS, staurosporine as a positive control). After 24 h, cells were collected for Western blotting with antibodies to the indicated proteins. To examine the levels of cytochrome c in the cytosol, mitochondria were separated *via* gradient centrifugation, and cell lysate without mitochondria was subjected for Western blotting with antibodies to the indicated proteins. The total cell lysate within intact mitochondria was used as the positive control. GDH, glutamate dehydrogenase. ***P < 0.001 by two-tailed Student’s *t*-test. ns, non significant. The densities of blots were analyzed with ImageJ software.

We ectopically expressed the plasmids encoding M and N proteins of SARS-CoV and SARS-CoV-2 in Vero E6 and HepG2 cells and examined their effects on apoptosis. The flow cytometry assays using Annexin V-Fluorescein 5-isothiocyanate (FITC)/propidium iodide (PI) double staining were performed to examine the apoptosis. As shown in **Figure 1A** and **Figure S1**, SARS-CoV-2 M significantly induced apoptosis in Vero E6 and HepG2 cells, whereas N showed no pro-apoptotic activity. Consistently, immunofluorescent analysis showed that the signals of cleaved-caspase-3 were only detected in cells expressing SARS-CoV-2 M but not N (**Figure 1B**). In contrast, SARS-CoV-encoded N showed pro-apoptotic activity (**Figure 1A**), indicating the different mechanisms by which these two viruses induce apoptosis. To further investigate N’s contribution to the SARS-CoV-2-induced apoptosis, we co-expressed SARS-CoV-2 M and N proteins in cells. Strikingly, the apoptosis rate was significantly enhanced compared to cells expressing M alone (**Figure 1C**). These results indicate that SARS-CoV-2 M but not N possesses the pro-apoptotic activity, while N can enhance the M-induced apoptosis.

In addition, we examined whether the stimulatory effect of SARS-CoV-2 N on the M-induced apoptosis is specific to M. We previously reported that SARS-CoV-2 ORF3a contains the apoptotic activity dependently of a caspase cascade (Ren et al., 2020). Thus, Vero E6 and HepG2 cells were ectopically expressed with SARS-CoV-2 ORF3a, N, or ORF3a plus N (ORF3a+N), respectively. Our data showed that the apoptosis rate was comparable in cells expressing ORF3a alone and ORF3a plus N (**Figure 1D**), indicating that SARS-CoV-2 N specifically enhances the M-induced apoptosis.

We sought to examine whether the apoptosis induced by SARS-CoV-2 M and the stimulatory effect of N on the pro-apoptotic activity of M is caspase-dependent. To this end, Vero E6 and HepG2 cells were ectopically expressed with SARS-CoV-2 M alone or M plus N (M+N) and treated with a general caspase inhibitor or specific inhibitor for caspase-8 or caspase-9. As shown in **Figures 1E, F**, all the caspase inhibitors effectively inhibited the apoptosis of cells expressing either SARS-CoV-2 M or M+N, indicating that the apoptosis induced by M or M+N depends on caspase activation.

Moreover, HEK293T cells were ectopically expressed with M, N, or M+N, and the activation of apoptosis cascade was examined *via* Western blotting of some apoptosis pathway components. Cells treated with staurosporine (STS), an apoptosis inducer, were used as the positive control. As shown in **Figures 1G, H**, SARS-CoV-2 M but not N induced the activation of caspase-8, caspase-9, and release of mitochondrial cytochrome c, confirming that the M-induced apoptosis is caspase-dependent and N alone does not possess the pro-apoptotic activity. Furthermore, the cleavages of caspase-8 and caspase-9, and the released cytochrome c from the mitochondrion, were unambiguously elevated in cells expressing M+N compared to those expressing M alone, suggesting that N indeed enhances the M-induced apoptosis and this effect is caspase-dependent.

Altogether, our findings indicate that SARS-CoV-2 M but not N induces caspase-dependent apoptosis, while N can specifically enhance the M-induced apoptosis.

### SARS-CoV-2 M Induces Apoptosis *via* Down-Regulating PDK1-PKB/Akt Signaling

**Figure 2:**
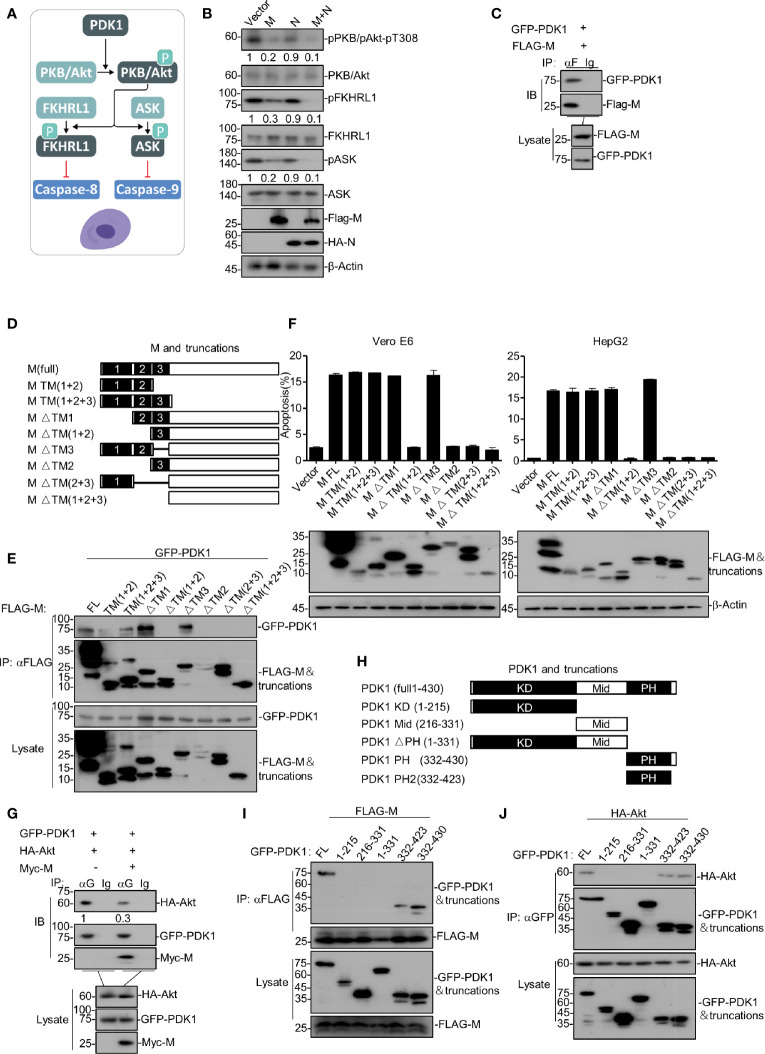
SARS-CoV-2 M induces apoptosis *via* down-regulating PDK1-PKB/Akt signaling. **(A)** A model of PDK1-PKB/Akt signaling associated with apoptosis. **(B)** HEK293T cells were transfected with FLAG-SARS-CoV-2 M, HA-SARS-CoV-2 N, and FLAG-SARS-CoV-2 M plus HA-SARS-CoV-2 N. 24 h later, cells were collected for Western blotting with antibodies to the indicated proteins. **(C)** HEK293T cells transfected with FLAG-SARS-CoV-2 M together with GFP-PDK1. After 24 h, cells were collected for co-immunoprecipitation (with anti-FLAG or IgG) and Western blotting (with anti-GFP or anti-FLAG). **(D)** Schematic representation of SARS-CoV-2 M and its truncations. **(E)** HEK293T cells transfected with FLAG-SARS-CoV-2 M and its truncations together with GFP-PDK1. After 24 h, cells were collected for co-immunoprecipitation (with anti-FLAG) and Western blotting (with anti-GFP or anti-FLAG). **(F)** Vero E6 and HepG2 cells were transfected with FLAG-SARS-CoV-2 M and its truncations. 24 h later, cells were collected for Western blotting (with anti-FLAG or anti-β-Actin) or stained with Annexin V-FITC/PI for flow cytometry analysis. The percentage of apoptotic cells was measured. **(G)** HEK293T cells transfected with FLAG-SARS-CoV-2 M and HA-Akt together with vector and GFP-PDK1, respectively. After 24 h, cells were collected for co-immunoprecipitation (with anti-GFP or IgG) and Western blotting (with anti-GFP, anti-Myc, or anti-HA). **(H)** Schematic representation of PDK1 and its truncations. **(I, J)** HEK293T cells transfected with GFP-PDK1 and its truncations together with FLAG-SARS-CoV-2 M and HA-Akt, respectively. 24 h later, cells were collected for co-immunoprecipitation (with anti-GFP) and Western blotting (with anti-GFP, anti-HA, or anti-FLAG). The densities of blots were analyzed with ImageJ software.

We sought to determine the mechanism of how SARS-CoV-2 M induces apoptosis. As illustrated in **Figure 2A**, PDK1-PKB/Akt signaling is an important anti-apoptotic pathway that inhibits caspases-8 and -9. A previous study reported that SARS-CoV M induces apoptosis in cells by interfering with PDK1-PKB/Akt signaling (Tsoi et al., 2014). We then examined whether the PDK1-PKB/Akt signaling pathway is related to the pro-apoptotic activity of SARS-CoV-2 M *via* Western blotting of the key components within PDK1-PKB/Akt cascade in HEK293T. As shown in **Figure 2B**, the expression of SARS-CoV-2 M attenuated the phosphorylated levels of PKB/Akt, FKHRL1, ASK, and the phosphorylated level of these proteins was reduced in cells expressing M+N compared to that of cells expressing M alone. These results indicate that SARS-CoV-2 M induces apoptosis *via* attenuating PDK1-PKB/Akt signaling, and the stimulatory effect of N on the M-induced apoptosis is also PDK1-PKB/Akt signaling-dependent.

Moreover, we examined how SARS-CoV-2 M interfered with PDK1-PKB/Akt signaling. Co-immunoprecipitation assays were performed with HEK293T cells expressing SARS-CoV-2 M and PDK1. As shown in **Figure 2C**, SARS-CoV-2 M was found to interact with PDK1. Moreover, we found that the activity of M impairing the PDK1-Akt interaction is dose-dependent (**Figure S2A**). Recent study on SARS-CoV-2 showed that M contains three N-terminal trans-membrane (TM1-3) domains, consisting of 222 amino acids (Gordon et al., 2020). To identify the SARS-CoV-2 M and PDK1 interaction domains, we constructed a serial of SARS-CoV-2 M truncations encoding different TM domains (**Figure 2D**) and ectopic expression of them in HEK293T cells together with PDK1. As shown in **Figure 2E**, domain-mapping assays indicated that the TM2 domain of SARS-CoV-2 M is essential for its interaction with PDK1.

To examine whether the interaction of SARS-CoV-2 M and PDK1 was required for the pro-apoptotic activity of M, we ectopically expressed M or its truncations in Vero E6 and HepG2 cells and conducted the FITC/PI double staining assays. As shown in **Figure 2F**, the SARS-CoV-2 M truncations that cannot interact with PDK1 were also unable to induce apoptosis, suggesting that the M-PDK1 interaction plays an essential role in M inducing apoptosis. Therefore, we proposed that the binding of SARS-CoV-2 M to PDK1 would interfere with the interaction between PDK1 and PKB/Akt, leading to the reduction of the downstream signaling events.

Furthermore, HEK293T cells were transfected with PDK1, Akt, and SARS-CoV-2 M plasmids, and co-immunoprecipitation assays were performed. Our results showed that PDK1-bound Akt was reduced in the presence of M (**Figure 2G**). Moreover, we generated a series of PDK1 domain fragments (**Figure 2H**) to map the domain of PDK1 responsible for its interaction with SARS-CoV-2 M or Akt. Our data showed that both M and Akt interacted with the PH domain of PDK1 (**Figures 2I, J**), suggesting that SARS-CoV-2 M can interfere with the Akt-PDK1 binding *via* targeting the PH domain of PDK1. In addition, we also identified that TM2 of M plays an essential role in blocking the PDK1-Akt interaction (**Figure S2B**).

Therefore, SARS-CoV-2 M induces apoptosis *via* its interaction with PDK1, which can attenuate PDK1-Akt interaction and then down-regulate PDK1-PKB/Akt signaling.

### SARS-CoV-2 N Facilitates M to Attenuate the PDK1-PKB/Akt Interaction Further

**Figure 3:**
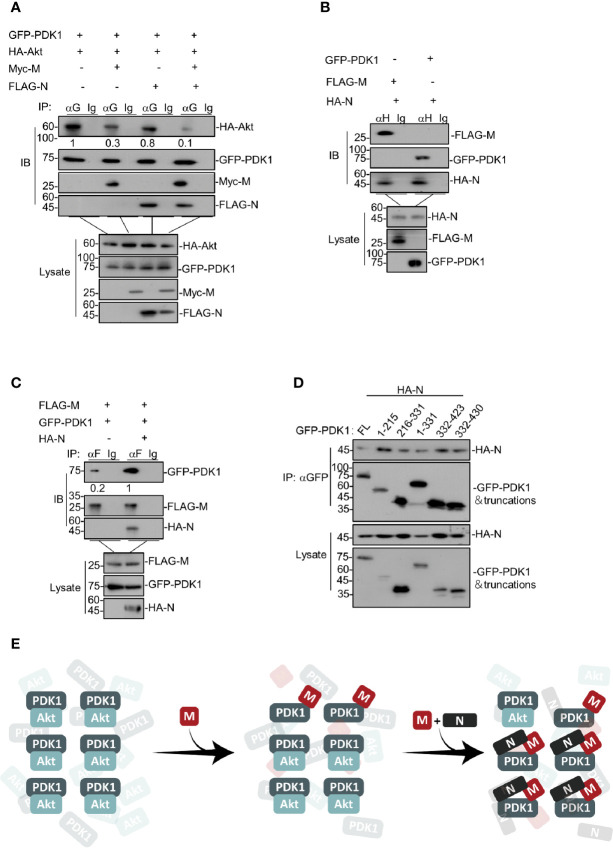
SARS-CoV-2 N facilitates M to attenuate the PDK1-PKB/Akt interaction further. **(A)** HEK293T cells were transfected with GFP-PDK1 and HA-Akt together with Myc-SARS-CoV-2 M, FLAG-SARS-CoV-2 N, and Myc-SARS-CoV-2 M plus FLAG-SARS-CoV-2 N, respectively. After 24 h, cells were collected for co-immunoprecipitation (with anti-GFP or IgG) and Western blotting (with anti-GFP, anti-Myc, anti-HA, or anti-FLAG). **(B)** HEK293T cells transfected with HA-SARS-CoV-2 N together with FLAG-SARS-CoV-2 M and GFP-PDK1, respectively. After 24 h, cells were collected for co-immunoprecipitation (with anti-HA or IgG) and Western blotting (with anti-GFP, anti-HA, or anti-FLAG). **(C)** HEK293T cells were transfected with FLAG-SARS-CoV-2 M and GFP-PDK1 together with vector and HA-SARS-CoV-2 N, respectively. After 24 h, cells were collected for co-immunoprecipitation (with anti-FLAG or IgG) and Western blotting (with anti-GFP, anti-HA, or anti-FLAG). **(D)** HEK293T cells transfected with GFP-PDK1 and its truncations together with HA-SARS-CoV-2 N. After 24 h, cells were collected for co-immunoprecipitation (with anti-GFP) and Western blotting (with anti-GFP or anti-HA). **(E)** A model for SARS-CoV-2 N facilitating M to attenuate the PDK1-PKB/Akt interaction further. The densities of blots were analyzed with ImageJ software.

After identifying that SARS-CoV-2 M induces apoptosis *via* competing with PKB/Akt for PDK1 interaction, we sought to determine how SARS-CoV-2 N enhances the M-induced apoptosis. To this end, we ectopically co-expressed PDK1, Akt, and M in the presence or absence of N in cells, followed by co-immunoprecipitation of PKD1 and its bound proteins. Our data showed that the presence of both M and N further reduced the level of Akt in the PDK1-immunocomplex compared with the effect of M alone (**Figure 3A**), indicating that SARS-CoV-2 N enhanced the effect of M to disrupt PDK1-PKB/Akt interaction.

We further examined the interactions between M and N, N and PDK1, respectively. Our results showed that SARS-CoV-2 N could interact with both M and PDK1 (**Figure 3B**). Moreover, N remarkably increased the protein level of M-bound PDK1 (**Figure 3C**), indicating that SARS-CoV-2 N can enhance the interaction between M and PDK1. Of note, although SARS-CoV-2 N was found to interact with PDK1, it showed the little effect to attenuate either the PDK1-PKB/Akt interaction (**Figure 3A**) or the phosphorylated level of PKB/Akt as M does (**Figure 2B**), implying that the interaction modes of M-PDK1 and N-PDK1 are different. Indeed, unlike the M-PDK1 interaction that only the PH domain of PDK1 can interact with M, N was found to interact with all the truncations of PDK1 (**Figure 3D**), suggesting a more flexible interaction mode for N and PDK1.

These results indicate that, as illustrated in **Figure 3E**, SARS-CoV-2 N can enhance the interaction between M and PDK1, which further attenuates the PDK1-PKB/Akt interaction crippled by M.

### SARS-CoV-2 N Enhances M-Induced Apoptosis *via* Simultaneously Interacting With M and PDK1

**Figure 4:**
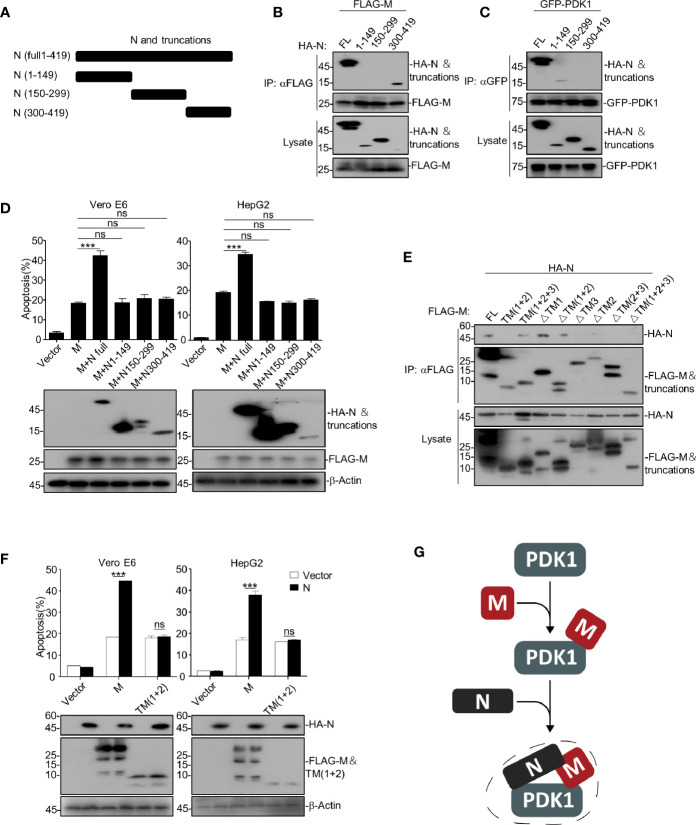
SARS-CoV-2 N enhances M-induced apoptosis *via* interacting with both M and PDK1. **(A)** Schematic representation of SARS-CoV-2 N and its truncations. **(B, C)** HEK293T cells transfected with HA-SARS-CoV-2 N and its truncations together with FLAG-SARS-CoV-2 M and GFP-PDK1, respectively. After 24 h, cells were collected for co-immunoprecipitation (with anti-FLAG or anti-GFP) and Western blotting (with anti-GFP, anti-HA, or anti-FLAG). **(D)** Vero E6 and HepG2 cells were transfected with FLAG-SARS-CoV-2 M together with HA-SARS-CoV-2 N and its truncations, respectively. After 24 h, cells were collected for Western blotting (with anti-FLAG, anti-HA, or anti-β-Actin) or stained with Annexin V-FITC/PI for flow cytometry analysis, and the percentage of apoptotic cells were measured. **(E)** HEK293T cells transfected with FLAG-SARS-CoV-2 M and its truncations together with HA-N. After 24 h, cells were collected for coimmunoprecipitation (with anti-FLAG) and Western blotting (with anti-FLAG or anti-HA). **(F)** Vero E6 and HepG2 cells were transfected with FLAG-SARS-CoV-2 M and FLAG-SARS-CoV-2 M TM(1 + 2) together with HA-SARS-CoV-2 N. After 24 h, cells were collected for Western blotting (with anti-FLAG, anti-HA, or anti-β-Actin) or stained with Annexin V-FITC/PI for flow cytometry analysis, and the percentage of apoptotic cells were measured. **(G)** A model for SARS-CoV-2 N enhancing the interaction of M and PDK1. ***P < 0.001 by two-tailed Student’s *t*-test. ns, non significant.

To uncover the detailed mechanism of how SARS-CoV-2 N enhances M-induced apoptosis, we constructed a serial of N truncations (**Figure 4A**) and examined their interactions with the full-length and truncations of M and PDK1, respectively. The domain-mapping plus co-immunoprecipitation assays showed that amino acid (a.a.) 1-149 were sufficient for binding to PDK1 (**Figure 4B**) and a.a. 300-419 of N were sufficient for binding to M (**Figure 4C**). In addition, our data showed that only the full-length N could enhance M-induced apoptosis (**Figures 4D**, **S3**), indicating that the enhancing effect of N on M-induced apoptosis requires the simultaneous binding of N to both M and PDK1. These results suggest that N interacts with M and PDK1 *via* different domains to form a stable complex and enhance M-induced apoptosis.

Furthermore, we found that the TM3 domain of M is required for the M-N interaction, but the TM (1 + 2) domain of M could not interact with N (**Figure 4E**). Consistently, although the TM (1 + 2) domain was sufficient to induce apoptosis, the TM (1 + 2)-induced apoptosis could not be further enhanced by N (**Figure 4F**). Of note, TM (1 + 2) domain was able to interact with PDK1 (**Figure 2E**). These results indicate that the domains within SARS-CoV-2 M critical for interacting with N (TM3) and PDK1 (TM2) are different, and the enhancing effect of N on M-induced apoptosis requires the interaction of M and N.

Together, our findings indicate that SARS-CoV-2 N can act as a scaffold to interact with both M and PDK1, thereby enhancing the interaction between M and PDK1 (**Figure 4G**), which further strengthens the M-mediated disruption of PDK1-PKB/Akt interaction and results in a more potent apoptotic induction than M alone.

### Interfering With the M-N Interaction Disrupts the Enhancing Activity of N on M-Induced Apoptosis

**Figure 5:**
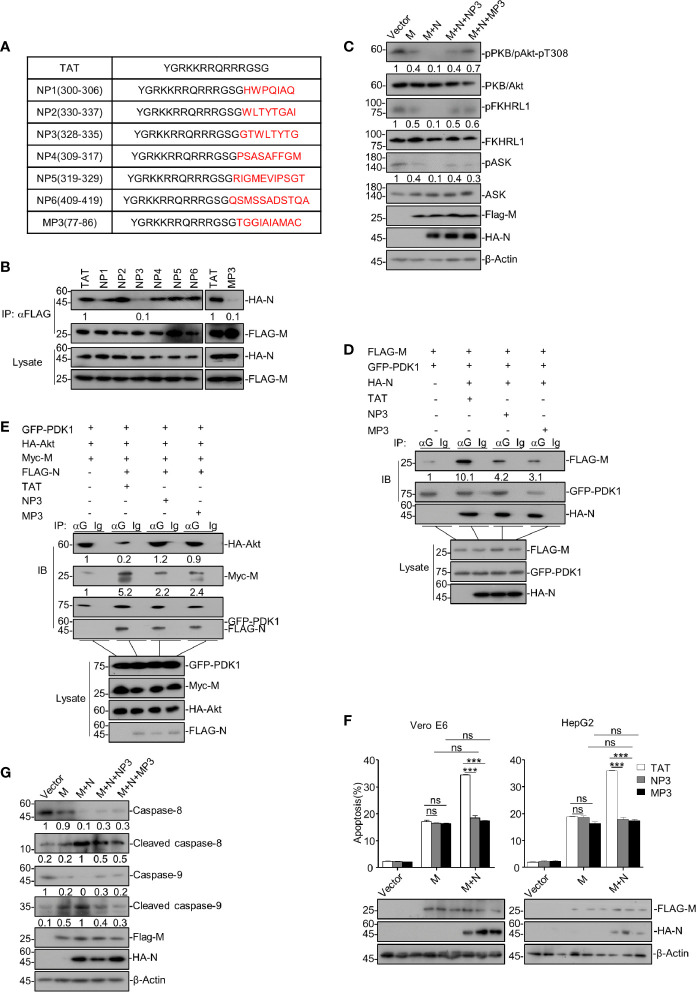
Disrupting the M-N interaction abolishes the enhancing activity of N on M-induced apoptosis. **(A)** The amino acid sequences of TAT, NP1-6, and MP3. **(B)** HEK293T cells were transfected with FLAG-SARS-CoV-2 M and HA-SARS-CoV-2 N and treated with TAT, NP1-NP6, and TM3, respectively. After 24 h, cells were collected for co-immunoprecipitation (with anti-FLAG) and Western blotting (with anti-FLAG or anti-HA). **(C)** HEK293T cells were transfected with FLAG-SARS-CoV-2 M and FLAG-SARS-CoV-2 M plus HA-SARS-CoV-2 N, respectively. Cells expressing FLAG-SARS-CoV-2 M plus HA-SARS-CoV-2 N were treated with NP3 and MP3, respectively. After 24 h, cells were collected for Western blotting with antibodies to the indicated proteins. **(D)** HEK293T cells were transfected with FLAG-SARS-CoV-2 M, GFP-PDK1 together with HA-SARS-CoV-2 N and treated with TAT, NP3, and TM3, respectively. After 24 h, cells were collected for co-immunoprecipitation (with anti-GFP or IgG) and Western blotting (with anti-FLAG, anti-HA, or anti-GFP). **(E)** HEK293T cells were transfected with GFP-PDK1, HA-Akt, Myc-SARS-CoV-2 M together with FLAG-SARS-CoV-2 N and treated with TAT, NP3, and TM3, respectively. After 24 h, cells were collected for co-immunoprecipitation (with anti-GFP, or IgG) and Western blotting (with anti-FLAG, anti-HA, anti-Myc, or anti-GFP). **(F)** Vero E6 and HepG2 cells were transfected with FLAG-SARS-CoV-2 M and SARS-CoV-2 M plus HA-SARS-CoV-2 N and treated with TAT, NP3, and TM3, respectively. After 24 h, cells were collected for Western blotting (with anti-FLAG, anti-HA, or anti-β-Actin) or stained with Annexin V-FITC/PI for flow cytometry analysis, and the percentage of apoptotic cells were measured. **(G)** HEK293T cells were transfected with FLAG-SARS-CoV-2 M and FLAG-SARS-CoV-2 M plus HA-SARS-CoV-2 N, respectively. Cells expressing FLAG-SARS-CoV-2 M plus HA-SARS-CoV-2 N were treated with NP3 and MP3, respectively. After 24 h, cells were collected for Western blotting with antibodies to the indicated proteins. ***P < 0.001 by two-tailed Student’s t-test. ns, non significant. The densities of blots were analyzed with ImageJ software.

As we have uncovered that SARS-CoV-2 N enhances the M-triggered apoptosis *via* its interactions with both M and PDK1, it would be intriguing to find out whether interfering with the M-N interaction would impair the enhancing effect of N. To this end, we designed 7 peptides, named NP1-NP6 and MP3, derived from and covered the amino acid sequences of a.a. 300-419 of SARS-CoV-2 N and the TM3 domain of M, respectively (**Figure 5A**), as these regions/domains within N and M were shown to be essential for the N-M interaction. Of note, these peptides are conjugated with TAT_47-57_, a commonly used cell-penetrating peptide (CPP), to increase their cell entry.

We then treated HEK293T cells expressing M and N with these peptides, followed by co-immunoprecipitation to assess M-N interaction. Our data showed that the peptides NP3 and MP3 effectively impaired the interaction between M and N, whereas other peptides and the negative control TAT showed no effect (**Figure 5B**).

We sought to examine whether these two peptides can reverse the attenuated PDK1-PKB/Akt signaling triggered by M together with N. Thus, HEK293T cells expressing M and N were treated with peptide NP3 or MP3. As shown in **Figure 5C**, NP3 or MP3 treatment remarkably increased the phosphorylation of PKB/Akt, FKHRL1, and ASK in cells expressing M and N. Moreover, the co-immunoprecipitation assays showed that N enhanced the M-PDK1 interaction, while NP3 or MP3 treatment effectively impaired the enhanced M-PDK1 interaction (**Figure 5D**). Furthermore, NP3 can work together with MP3 to confer a stronger inhibitory effects on PDK1-M interaction (**Figure S4**). Besides, the attenuated interaction between PDK1 and Akt in cells expressing M+N was effectively restored by NP3 or MP3 treatment (**Figure 5E**).

Moreover, the apoptosis rate of M+N-expressing cells treated with either NP3 or MP3 was comparable with that of cells expressing M alone in the absence of NP3 or MP3 treatment (**Figure 5F**), indicating that NP3 or MP3 abolished the enhancing effect of N on the M-induced apoptosis. Neither of these two peptides showed any effect on the M-induced apoptosis in N’s absence, confirming that they specifically target the M-N interaction. Besides, NP3 or MP3 treatment remarkably reduced the cleaved levels of caspase-8 and caspase-9 in M+N-expressing cells (**Figure 5G**).

Overall, our findings suggest that interrupting the M-N interaction can specifically block the enhancing effect of N on the M-induced apoptosis by restoring the attenuated PDK1-PKB/Akt signaling.

## Discussion

**Figure 6:**
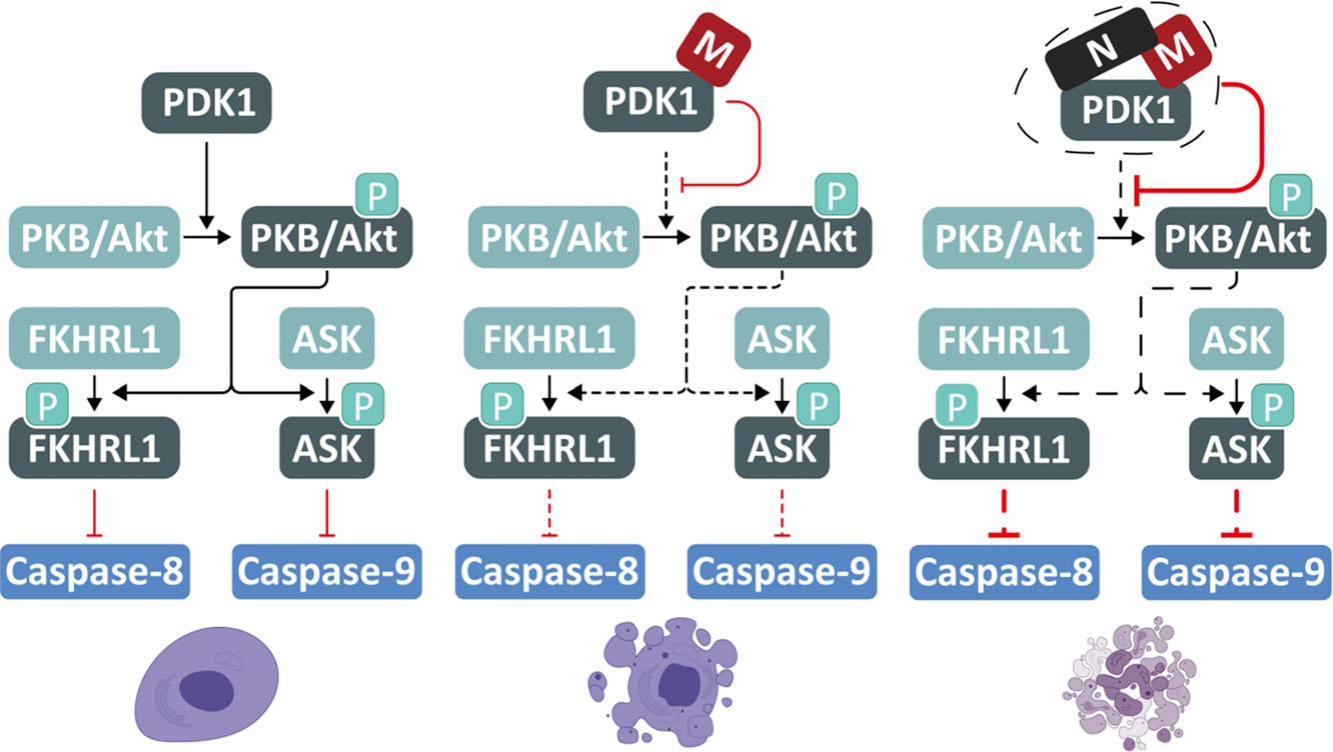
A proposed model for SARS-CoV-2 M and N to induce apoptosis. SARS-CoV-2 M possesses a pro-apoptotic activity *via* interacting with PDK1 to attenuate PDK1-PKB/Akt interaction and signaling, while N acts as a scaffold for M and PDK1 to enhance the M-induced apoptosis.

Given the great threat of the COVID-19 pandemic, a better understanding of SARS-CoV-2 is urgently needed. Herein, we uncovered that SARS-CoV-2 M possesses a pro-apoptotic activity *via* interacting with PDK1 to attenuate PDK1-PKB/Akt interaction and signaling, whereas N functions as a scaffold for M and PDK1 to promote the M-induced apoptosis (**Figure 6**). These findings demonstrate the sophisticated regulation of apoptosis by SARS-CoV-2.

The PDK1-PKB/Akt signaling pathway is essential for cell survival and is manipulated by viruses to induce apoptosis and facilitate viral replication (Soares et al., 2009). Through its PH domain, PDK1 facilitates PKB/Akt phosphorylation and stimulates the activity of PKB/Akt, allowing it to phosphorylates its downstream substrates, including FKHRL1 and ASK. This study found that SARS-CoV-2 M could competitively interact with the PH domain of PDK1 *via* its TM2 domain, preventing the interaction between PKB/Akt and PDK1, thereby attenuating the phosphorylation and kinase activity of PKB/Akt. When the activity of PKB/Akt is inhibited, the phosphorylation of FKHRL1 and ASK are congruently reduced, resulting in inhibiting downstream caspases-8 and -9. The conserved pro-apoptotic activity of M encoded by different coronaviruses indicates its functional importance in the coronaviral life cycle and pathogenicity. Moreover, the truncates of SARS-CoV-2 M that contain the essential PDK1-binding domain TM2 can induce apoptosis, implying that the interaction between the TM2 domain of M and the PH domain of PDK1 is sufficient to prevent the downstream substrate of PDK1 from accessing the key enzymatic sites of PDK1.

Although the amino acid sequence of SARS-CoV-2 N is conserved with its counterpart of SARS-CoV, our findings show that SARS-CoV-2 N has no pro-apoptotic activity, as its ectopic expression alone causes neither cell death nor the cleavage of caspase-8/-9 and the release of cytochrome c, which is the hallmark of caspase-dependent apoptosis. Surprisingly, we discovered that SARS-CoV-2 N could enhance the M-induced apoptosis by reducing the attenuated PDK1-PKB/Akt signaling triggered by M. Our domain-mapping and co-immunoprecipitation assays identified that SARS-CoV-2 N could interact with both M and PDK1 *via* different domains. Under such a condition, N can functions as a scaffold to strengthen the interaction between M and PDK1, thereby enhancing M′s activity to compete with PKB/Akt for PDK1 interaction. As a result, SARS-CoV-2 N can function as an agonist cofactor of M to facilitate M’s pro-apoptotic activity, representing a novel mechanism by which SARS-CoV-2 promotes apoptosis.

Apoptosis is an essential host-defense process that controls the viral infection and regulates inflammatory responses (Roulston et al., 1999). Moreover, virus-induced apoptosis is tightly associated with viral pathogenicity and pathogenesis (Martin et al., 2012). Many viruses encode more than one protein to trigger apoptosis. For example, Enterovirus 71-encoded 2A, 2B, and 3C all trigger apoptosis *via* different mechanisms (Kuo et al., 2002; Cong et al., 2016; Li et al., 2017). Interestingly, we recently demonstrated that SARS-CoV-2 ORF3a induces apoptosis *via* a caspase-dependent extrinsic pathway. The current study uncovers that SARS-CoV-2 M shows the pro-apoptotic activity, which can be enhanced by N. The discovery that SARS-CoV-2 encodes multiple proteins with pro-apoptotic or regulatory activities highlights the importance of SARS-CoV-2-induced apoptosis for COVID-19 pathogenesis. A transcriptome study has consistently shown that SARS-CoV-2 induced apoptosis in lymphocytes, which may be one of the leading causes of patients’ lymphopenia (Xiong et al., 2020).

Moreover, as aforementioned, the pro-apoptotic activity was only shown for SARS-CoV-2 M but not N. Currently, we found that SARS-CoV-2 N is the agonist cofactor of the M-induced apoptosis, and it can only enhance the M-induced apoptosis but not ORF3a-triggered apoptosis, indicating the specificity of N regulating apoptosis. The difference in the pro-apoptotic mechanism probably contributes to the difference in the pathogenicity of these two coronaviruses. Besides, our findings suggest that the mode of SARS-CoV-2 N enhancing the M-induced apoptosis may be more conducive for it to control virus-induced apoptosis flexibly, which probably provide certain advantages for SARS-CoV-2 to avoid host immune response at the early stage of infection.

The protein-protein interaction regions are attractive targets for drug development. We did not design and test the peptides targeting the PDK1-M or PDK1-N interaction because blocking the PDK1-binding sites to M or N or designing peptides derived from PDK1 could disrupt the normal structures and functions of PDK1 that mediates multiple downstream substrates critical for cell survival. Instead, we show that the rationally designed peptides targeting the binding regions between M and N can effectively disrupt the M-N interaction, leading to restoring the PDK1-PKB/Akt signaling attenuated by M and N.

In summary, the findings in this study revealed a novel mechanism by which SARS-CoV-2-encoded M triggers apoptosis with the help of N, which expands our understanding of the two key structural proteins of SARS-CoV-2, and sheds light on the pathogenicity of this life-threatening virus.

## Data Availability Statement

The original contributions presented in the study are included in the article/**Supplementary Material**. Further inquiries can be directed to the corresponding authors.

## Author Contributions

YR performed the experiments with the help of AW, YF, TS, DW, CW, MH, JM, LJ, and YR, WZ, YQ, and XZ designed the experiments. YR, YQ, and XZ interpreted the results and wrote the manuscript. All authors contributed to the article and approved the submitted version.

## Funding

This work was supported by the Strategic Priority Research Program of CAS (XDB29010300 to XZ), National Science and Technology Major Project (2018ZX10101004 to XZ), National Natural Science Foundation of China (31970169 to XZ, 81873964 to YQ and 32000131 to DW), China Postdoctoral Science Foundation (2019M660198 to YR) and Grant from the CAS Youth Innovation Promotion Association (2020332 to YQ).

## Conflict of Interest

The authors declare that the research was conducted in the absence of any commercial or financial relationships that could be construed as a potential conflict of interest.

## Publisher’s Note

All claims expressed in this article are solely those of the authors and do not necessarily represent those of their affiliated organizations, or those of the publisher, the editors and the reviewers. Any product that may be evaluated in this article, or claim that may be made by its manufacturer, is not guaranteed or endorsed by the publisher.
