# Supplementary material for: SARS-CoV-2 Membrane Glycoprotein M Triggers Apoptosis With the Assistance of Nucleocapsid Protein N in Cells
Source: Front Cell Infect Microbiol. 2021 Aug 25;11:706252. doi: 10.3389/fcimb.2021.706252 (PMC8425412; doi:10.3389/fcimb.2021.706252)
Supplement: Supplementary file 1 [file DataSheet_1.pdf]

Figure S1

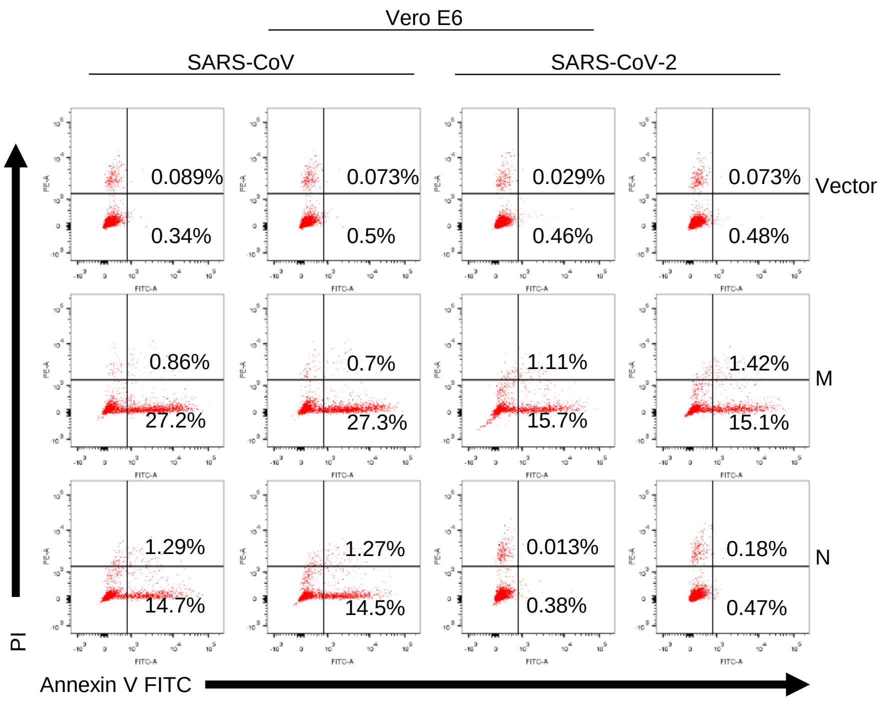

Figure S1. Vero E6 cells were transfected with vector, FLAG-SARS-CoV-2 M or FLAG-SARS-CoV-2 N. After 24h, cells were treated with Annexin V-FITC/ PI for flow cytometry analysis.

Figure S2

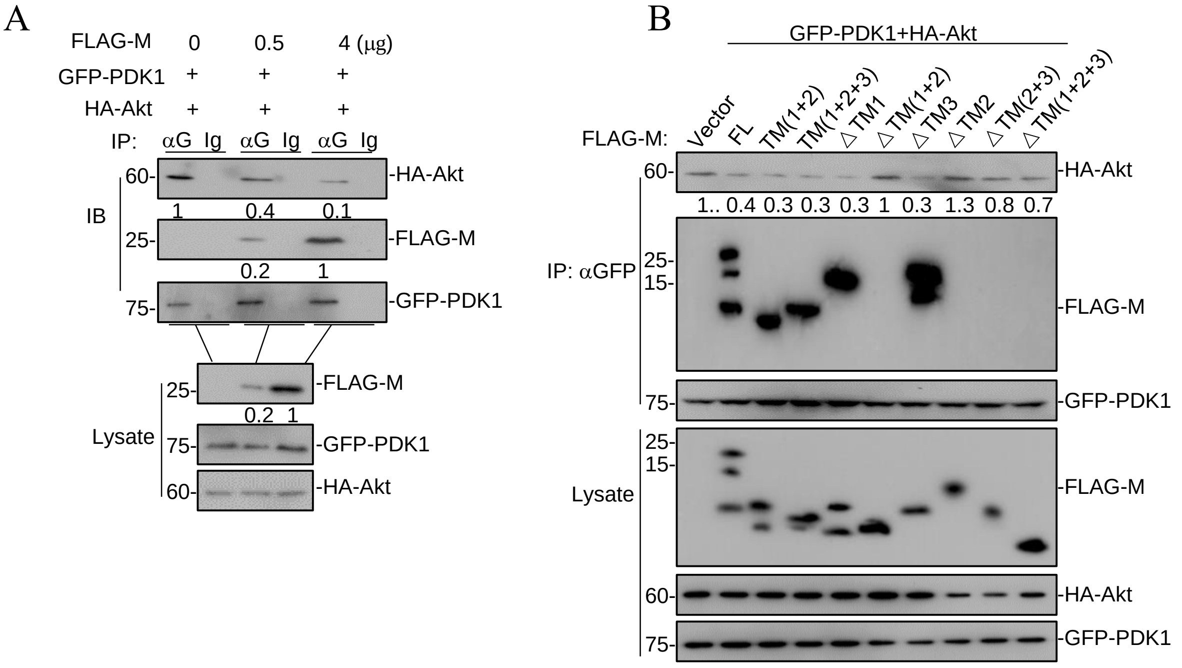

Figure S2. (A) HEK293T cells transfected with GFP-PDK1 and HA-Akt together with vector or FLAG-SARS-CoV-2 M (0.5μg or 4μg). After 24h, cells were collected for co-immunoprecipitation (with anti-GFP or IgG) and western blotting (with anti-GFP, anti-HA or anti-FLAG). **(B)** HEK293T cells transfected with GFP-PDK1 and HA-Akt together with vector or FLAG-SARS-CoV-2 M and its mutants. After 24h, cells were collected for co-immunoprecipitation (with anti-GFP or IgG) and western blotting (with anti-GFP, anti-HA or anti-FLAG). The densities of blots were analyzed with ImageJ software.

Figure S3

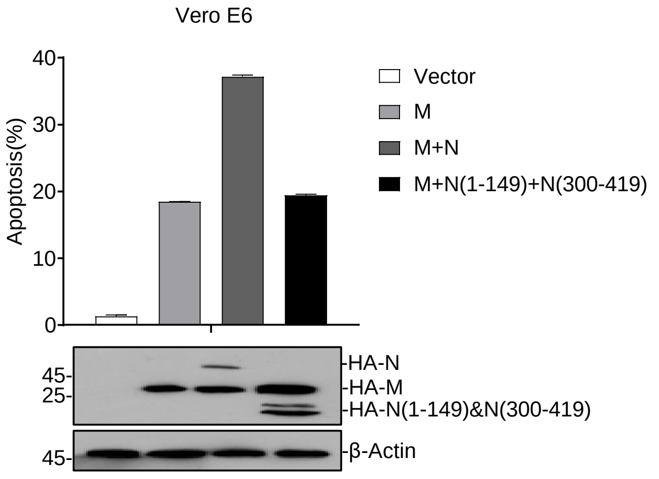

Figure S3. Vero E6 cells transfected with vector, HA-SARS-CoV-2 M together with HA-SARS-CoV-2 N or with HA-SARS-CoV-2 N(1-149)+N(300-419). After 24h, cells were stained with Annexin V-FITC/ PI for flow cytometry analysis, and the percentage of apoptotic cells were measured.

Figure S4

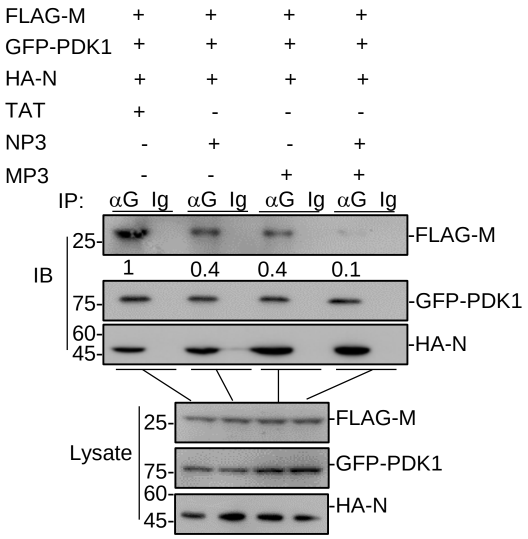

Figure S4. HEK293T cells transfected with GFP-PDK1, HA-Akt together with FLAG-SARS-CoV-2 M and treated with TAT, NP3, TM3 and NP3+MP3, respectively. After 24h, cells were collected for co-immunoprecipitation (with anti-GFP or IgG) and western blotting (with anti-GFP, anti-HA or anti-FLAG). The densities of blots were analyzed with ImageJ software.
